# Root xylem plasticity to improve water use and yield in water-stressed soybean

**DOI:** 10.1093/jxb/erw472

**Published:** 2017-01-07

**Authors:** Silvas J. Prince, Mackensie Murphy, Raymond N. Mutava, Lorellin A. Durnell, Babu Valliyodan, J. Grover Shannon, Henry T. Nguyen

**Affiliations:** 1 University of Missouri, Division of Plant Sciences, Columbia, MO 65211, USA

**Keywords:** Drought stress, nested association mapping, pod harvest index, pod retention, root anatomy, soybean (*Glycine max*), yield protection under drought.

## Abstract

We tested the hypothesis that increasing the number of metaxylem vessels would enhance the efficiency of water uptake in soybean (*Glycine max*) and decrease the yield gap in water-limited environments. A panel of 41 soybean accessions was evaluated in greenhouse, rainout shelter, and rain-fed field environments. The metaxylem number influenced the internal capture of CO_2_ and improved stomatal conductance, enhancing water uptake/use in soybeans exposed to stress during the reproductive stage. We determined that other root anatomical features, such as cortex cell area and the percentage of stele that comprised cortical cells, also affected seed yield under similar growth parameters. Seed yield was also impacted by pod retention rates under drought stress (24–80 pods/plant). We surmise that effective biomass allocation, that is, the transport of available photosynthates to floral structures at late reproductive growth stages (R6–R7), enables yield protection under drought stress. A mesocosm study of contrasting lines for yield under drought stress and root anatomical features revealed that increases in metaxylem number as an adaptation to drought in the high-yielding lines improved root hydraulic conductivity, which reduced the metabolic cost of exploring water in deeper soil strata and enhanced water transport. This allowed the maintenance of shoot physiological processes under water-limited conditions.

## Highlight

Altering root xylem developmental plasticity by increasing the number of vessels and decreasing their diameter enhances water uptake and reduces yield loss in soybean under water-limited conditions.

## Introduction

Global crop production is hampered by drought stress, a major constraint (Schmidhuber and Tubiello, 2007) that increases the risks associated with rain-fed agriculture (Lobell *et al.*, 2011). The US accounts for 38% of global soybean (*Glycine max*) production, with annual yields reaching 85 metric tons (Grassini *et al.*, 2015). The US is predicted to increase its use of soybean meal and oil by 20% and 30%, respectively, with US-produced soybean accounting for 20% of world trade (Wescot and Hansen, 2016). Modifying the root system architecture has been proposed as a pipeline to achieve a second green revolution and enhance crop yield (Den Herder *et al.* 2010). Root traits play a significant role in crop productivity in soils lacking water and nutrients (Bengough *et al.* 2011). For example, in the drought scenario, plants with deeper roots are able to acquire water from deeper soil domains (Hammer *et al.*, 2009). Because yield is influenced by many factors, including phenotype and its crosstalk with the environment, it cannot be used directly as a selection criterion in crop breeding programmes (Chimungu *et al.*, 2014*b*). Integrating root architectural, anatomical, and physiological phenes will facilitate the breeding of a crop ideotype for drought conditions (Lynch, 2011).

Multiple studies in maize (*Zea mays*) have reported that root cortical cells and cortical aerenchyma confer improved plant performance in nutrient-limited soils, including those with limited phosphorus (Fan *et al.*, 2003; Lynch, 2011), nitrogen (Saengwilai *et al.*, 2014), and water availability (Zhu *et al.*, 2010; Jaramillo *et al.*, 2013; Chimungu *et al*., 2015*b*). Root cortical cell size (CCS) and file number have been associated with deeper soil exploration and greater water acquisition, and thus enhanced drought tolerance in maize (Chimungu *et al.*, 2014*a*, *b*; Chimungu *et al.*, 2015*b*), providing higher biomass and grain yields (Chimungu *et al.*, 2014*a*, *b*). Of the studies root phenes, stele diameter was observed to be a better predictor of root penetrative ability than root diameter (Chimungu *et al*., 2015*a*). A recent comparative study of rice (*Oryza sativa*) and wheat (*Triticum aestivum*) by Kadam *et al.*, 2015 revealed that stele and xylem number/diameter were more responsive to water-deficit conditions in wheat than in rice owing to xylem developmental plasticity. Similar interspecies variation in xylem diameter was observed in a drought study across a single genotype of six different legume crops compared with pearl millet (*Pennisetum glaucum*) (Purushothaman *et al.*, 2013).

A recent study in different rain-fed environments varying in soil type and climatic factors revealed that soybean genotypes with better modulation of shoot and root traits were more productive upon exposure to drought stress in the reproductive stage (Prince *et al.*, 2016). This study also detailed the natural genetic ability of exotic (landraces not of US origin) species to gain access to water and nutrients by enhancing their lateral root angle, a functional trait with the ability to improve grain production in water-limited environments. However, in soybean there is a yield gap, which is the difference between the existing yield potential of a given variety and on-farm yield patterns (Van Roekel and Purcell, 2014). This yield gap changes dramatically with water availability during a cropping season (Grassini *et al.*, 2014*a*; Grassini *et al.*, 2015). Identifying genotypes with effective water uptake and use efficiency in both rain-fed and irrigated fields is critical to narrow this yield gap.

This study builds on the earlier studies that indicated that metaxylem properties (number and diameter) are associated with water-use efficiency in cereal crops (Kadam *et al.*, 2015), with an increase in hydraulic resistance enhancing yield under drought stress (Richards and Passioura, 1989). Another study with legumes such as common bean (*Phaseolus vulgaris*), cowpea (*Vigna unguiculata*), and soybean showed that these crops possess a moderate number of xylem vessels and thicker roots that are adapted to rainy seasons (Purushothaman *et al.*, 2013). However, the relationship between root xylem morphology and yield under drought stress has yet to be established in legumes. Thus, our aim in this study was to test the hypothesis that metaxylem plasticity enhances water uptake and improves plant performance to protect yield under water stress. Diverse, high-yielding soybean genotypes (including cultivar and plant introductions) with contrasting numbers of metaxylem elements (MXs) were evaluated under water-stress and well-watered conditions in controlled environments and in field conditions.

## Materials and methods

Forty-one high-yielding germplasms selected for a soybean nested association mapping (NAM) project [32 elite lines, 8 plant introductions (PIs), and a hub parent (IA3203)] (see Supplementary Table S1 at *JXB* online) (Anderson *et al.*, 2014) were studied in four experiments. Two experiments were conducted in the greenhouse and two experiments were conducted in the field during the years 2012 and 2013.

### Greenhouse trial 1 (GHT1): seedling stage root architectural study

Plants were grown in the Ernie and Lotti Sears Plant Growth Facility, University of Missouri, Columbia, MO, USA, under a 14/10-h light/dark photoperiod with supplemental lamps that maintained 1400 klx and a day/night temperature of 26.7/23.9°C ± 2°C. Ten seeds were sown into a homogenized mix of 2:1 Turface MVP (Hummert International, St. Louis, MO, USA) and medium grit sand (Quikrete, Columbus, OH, USA) in D60H Deepots (983 mL capacity; Stuewe & Sons Inc., Tangent, OR, USA). Deepots were dampened twice daily until germinating seeds had reached the V0 (open cotyledon) stage; plants were then watered once daily with enough excess liquid to flow through the growth media. Five root systems were carefully removed from the growth media at the V1 growth stage (first fully expanded trifoliate leaf) to study the root system traits. Before scanning the root systems, the roots were stored in 70% ethanol, which does not cause any significant alterations to root characteristics. Root systems were individually placed in a 14 × 30 × 2 cm Plexiglas tray (Regent Instruments Inc., Quebec, Canada) filled with distilled water to a depth of 1 cm to allow for the separation of fine roots prior to image capture. The tray was then placed on a large-field flatbed scanner (Epson 6500 XL, Epson America, Inc., Long Beach, CA, USA). Images were analysed using WinRHIZO Pro 2009 software (Regent Instruments Inc.).

### Greenhouse Trial 2 (GHT2): root elongation zone microscopy analysis

**Fig. 1.**
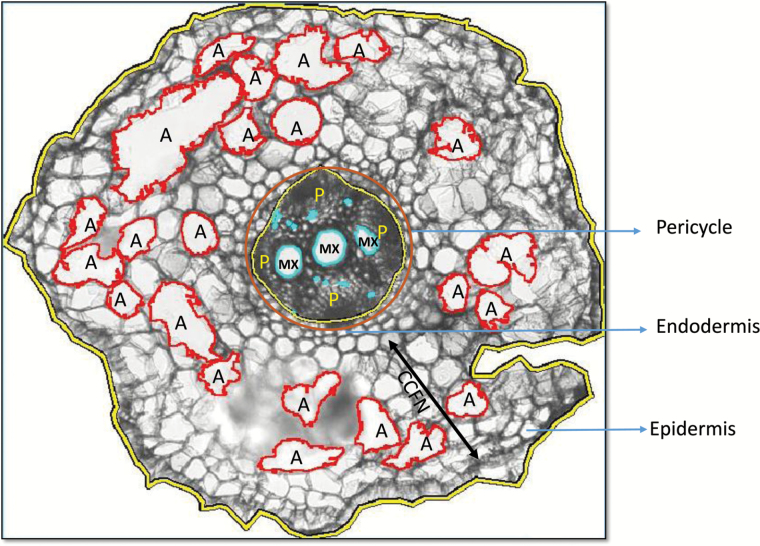
Root anatomical analysis of soybean LG05-4317 using Rootscan software with various root anatomical features shown. A: aerenchyma; CCFN: cortical cell file number; P: phloem; MX: metaxylem.

Plants were grown in a growth chamber set to the same environmental conditions and in the same soil conditions detailed in GHT1. The growth zones of the taproot were experimentally determined for the soybean reference genotype Williams 82 (PI 518671) grown in the same growth chamber under identical conditions in a replicated trial. The three zones of Williams 82 genotype taproot–—meristem, elongation, and mature zones—constituted a ratio of 1:2:3 of total taproot length, respectively. The 2 cm flanking the point located at 50% of each taproot’s length were collected for anatomical analysis; tissue was collected from five biological replicates and stored in FAA fixative (90 mL of 70% ethanol to 5 mL of glacial acetic acid to 5 mL of 38% formaldehyde) at 4°C until embedding was carried out. One centimetre of tissue removed from each replicate and embedded into 5% MetaPhor agarose using plastic moulds. Cross sections were made (~50–60 µm thickness) using the Vibratome 3000 Plus (Leica, Wetzlar, Germany). Wet-mount slides were prepared with 10 samples per replicate and stained with 0.05% toluidine blue for added contrast of the cell walls. Ten subsamples from all replicates were selected for image analysis after inspection for breakage and uniformity. The selected cross sections were imaged using a Leica DM5500 inverted light microscope (Leica) and Leica DFC290 colour camera at an objective magnification of ×10. Images were analysed using RootScan2 (Burton *et al.*, 2012) and the root anatomical features of LG05-4317 soybean are shown in Fig. 1.

### Field Trial 1 (FT1): evaluation of NAM lines for reproductive stage-specific drought stress

FT1 was conducted at the Bradford Research and Extension Center (BREC) in Columbia, MO, USA (38°53’50.0”N and 92°13’05.0”W) under an automated rainout shelter. The 41 lines were grown in a randomized complete block design in two replicate plots of 8-foot (2.44 m) rows with standard 30-inch (76.2 cm) row spacing in both water-stressed plots (under the rainout shelter) and irrigated plots (using a drip irrigation system bi-weekly for 18 h to keep the soil moisture at an optimal level). The rainout shelter would move into position to cover the plots when triggered by 0.01 inches (0.25 mm) of rain and return to its resting position after rain events had occurred. The experiment lasted from May to August 2012 and experienced severe drought during June and July (Supplementary Table S2). The water available to the plants were measured using a Delta-T moisture PR2 probe at four different depths of 300 mm, 400 mm, 600 mm and 1000 mm (Supplementary Fig. S1). Canopy wilting was measured as visual symptoms on a 1–5 scale (1: >20% of plot exhibits wilting to 5: >90% of plot exhibits wilting; Supplementary Fig. S2). Gas exchange measurements were taken from three individuals per plot bi-weekly for the water-limited and control plots using the LI-6400XT (LI-COR Biosciences, Lincoln, NE, USA) at three time points [early vegetative (V4), early reproductive (R1–R2), and mid-seed filling (R5–R6) stages]. Leaf area measurements on the third fully expanded trifoliate leaf were conducted using the Handheld Laser Leaf Area Meter C-203 (CID Biosciences, Camas, WA, USA) in both treatment plots after plants had reached the R5 growth stage.

### Field Trials 2 and 3 (FT2 and 3): evaluation of biomass partitioning, allocation, and water-use efficiency

FT2 and 3 were conducted from May to August 2013 at two locations with varying climatic conditions and soil type: FT2 was conducted at the BREC in Columbia, MO, USA (38°53’50.0”N and 92°13’05.0”W) and FT3 was conducted at the Rhodes Memorial Research Farm (Delta Center) in Clarkton, MO, USA (36°29’26.1”N and 89°57’42.3”W). BREC field conditions are characterized by clay soils and a thick clay pan at a depth of 12–24 inches (30.5–61.0 cm). Field plots at the Delta Center are characterized by deep, sandy loam soils that are uniform. Both irrigated and rain-fed plots were maintained in FT2 and FT3. Details on precipitation level, irrigation level, and average maximum daily temperature of the field locations are provided in Supplementary Table S2.

Replicated 2.5 m rows were planted in a randomized complete block design for the stress and control treatments at both locations. The control plots were irrigated as per the Woodruff Irrigation Chart calibrated to the location and ‘maturity group’. Shoot biomass was measured at two time points in the stress and control plots in FT 2: R2–R3 (pod filling) and R6–R7 (fully mature). By studying the allocation of biomass to reproductive structures such as flowers and pods at two different stages under rain-fed field conditions, we were able to examine the genotypic variability in reproductive structure retention, which is crucial for enhancing productivity. To this end, two representative plants per plot were removed and weighed, then placed in an oven at 60°C for 72 h before being weighed again. The plot yield was collected at both field locations. Twenty seeds from each plot at BREC were ground for carbon and nitrogen isotopic analysis. Ground seed and leaf samples (200 mg) were sent to the Stable Isotope Mass Spectrometry Laboratory at Kansas State University (Manhattan, KS, USA) for carbon and nitrogen analysis.

### Greenhouse trial 3 (GHT 3): detailed mesocosm study with genotypes contrasting for yield under stress

Finally, six lines were selected from each of the high-yield selection (HYS) and low-yield selection (LYS) groups from previous screenings of 40 lines in field conditions with an emphasis on the yield components from experiments FT1 and FT2, the rain-fed single plot yield and the rain-fed 8-foot row yield, respectively. Selected lines were grown in 5-gallon (22.73 L) poly-containers (Hummert International, Earth City, MO, USA) in a growth media described by Chimungu *et al.* 2014*a*. Fifty grams of Osmocote 14-14-14 NPK time-release fertilizer (The Scotts Miracle-Gro Company, Marysville, OH, USA) was incorporated into each pot before sowing. The experiment was conducted in the greenhouse located at the University of Missouri campus between November 2015 and January 2016. The photoperiod was set to 14/10 h (day/night), with day/night temperatures maintained at 27.8/24.4°C ± 2°C. Three replicates were grown for both drought and control treatments and five seeds were germinated. The single best seedling per pot was selected at V0. Water was withheld from the drought-treated pots for 28 days after plants reached the R1 growth stage. Field capacity was maintained for the control pots. Gas exchange, leaf area, and visual leaf wilting symptoms were measured after 28 days of stress treatment. Root and shoot hydraulic flows were measured using the Dynamax HCFM-XP Generation 3 (Dynamax Inc., Houston, TX, USA) with a quasi steady state method, immediately before the roots were harvested for sectioning. Ten centimetres of taproot basal to the root–shoot junction and 10–20 cm of the attached lateral roots were harvested, washed, and stored in FAA at 4°C until sectioning was carried out. Sections were made using the protocol of Burton *et al.* (2012), followed with analysis using RootScan 2 software. Images were captured using a DFC 290 colour camera on a Zeiss Axiovert 200M (Carl Zeiss AG, Oberkochen, Germany), using the automatic stitching feature at ×5 magnification. Five images of each replicate were selected for the analysis; images with intact epidermal layers and uniform section thickness were selected, and the overall image clarity was considered. Image analysis was completed using RootScan2 (Burton *et al.*, 2012. The metaxylem area was converted into metaxylem diameter (mm) assuming circularity of vessels as detailed in Smith *et al.* (2013).

## Results

### Root vigour at early vegetative stage

The diverse NAM panel lines exhibited high phenotypic variation for root architectural traits measured in this study (Supplementary Table S1). We observed significant variation (Supplementary Table S3, available at *JXB* online) among global root architectural traits (*P* < 0.0001) evaluated in GHT1. However, no significant variations were identified for shoot phenotypic values. In this study, the exotic PI 427136 showed maximum phenotypic variations for the root projected area and surface area compared to the elite lines. In contrast, the root traits of the elite lines LG00-3372 and 5M20-2-5-2 exceeded all trait values among the exotic PIs; for example, the total root length and the number of crossings had maximum phenotypic values of 1210.88 cm and 783.40 cm, respectively. Similarly, several traits of the elite line S06-13640, including root volume, number of forks, and number of tips had values greater than those traits in the PIs (Supplementary Table S1). The elite line LG00-3372 and PI 427136 showed higher phenotypic values for total root length (Supplementary Fig. S3, available at *JXB* online), whereas the exotic PI 574486 and elite line LD01-5907 had the lowest phenotypic values for total root length. These phenotypic variations for root architectural traits in seedling stage are vital and offer the potential to improve plant performance through breeding programmes.

### Radial root anatomy

**Fig. 2.**
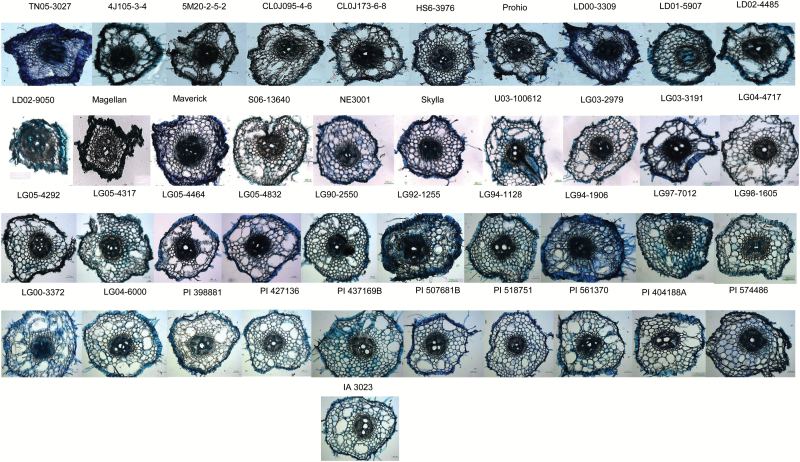
Natural genetic variation of root anatomical features observed in 41 soybean lines of the soybean NAM panel. Scale bar = 100 μm.

In cereal crops, root cortex cell-related traits such as thickness, cell count, cell wall area, and distal cell size are strong predictors of root bending to facilitate penetration into hard soil layers. We found significant variation for these root anatomical traits among the elite and PI accessions in the early seedling stage studied in GHT2 (Fig. 2; Supplementary Table S4, available at *JXB* online). In this study, the LD02-9050 line had the lowest percentage of root cross-sectional area (RXSA) with a total cortex area (TCA) of 14.6%, an average cortical cell number of 96 and a cortical cell file number (CCFN) of 4 (Supplementary Table S4). In elite line S06-13640, 31.6% of the TCA was composed of an average of 674 cortical cells with a CCFN of 11.7. Positive correlations were observed between cortical cell number and TCA (R^2^ = 0.66), and between CCFN and TCA (R^2^ = 0.72). Total stele areas in the study ranged from 0.03 mm^2^ (LD02-9050) to 3.66 mm^2^ (5M20-2-5-2). The percentage of xylem vessels in the stele ranged from 0.4% in NE3001 to 8.8% in 4J105-3–4. The total stele areas correlated positively with MX (R^2^ = 0.579, *P* < 0.01) and correlated negatively with the TCA (R^2^ = −0.32; *P* < 0.05) and CCFN (R^2^ = −0.39; *P* < 0.05).

### Drought-adaptive shoot physiological mechanisms

**Fig. 3.**
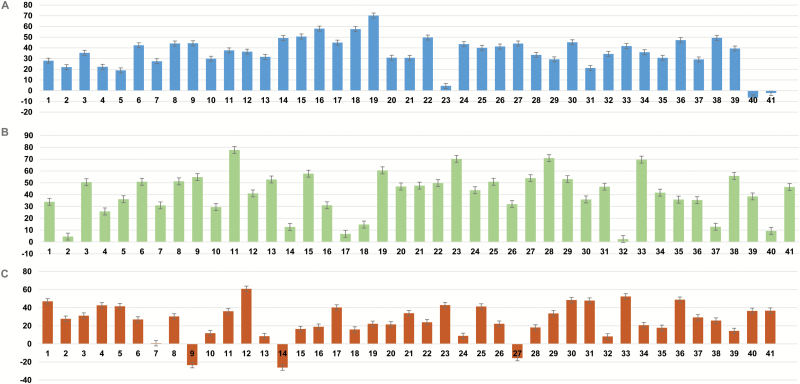
Reductions observed during FT1 for variations in (**A**) pod number, (**B**) stomatal conductance, and (**C**) leaf area in NAM lines selected in this study.

We recorded a significant reduction in stomatal conductance, leaf area, and pod number (Table 1; Fig. 3) in FT1 under the rainout shelter (see Supplementary Fig. S4A at *JXB* online) with imposed water-deficit stress. Genotype PI 427136 maintained higher pod retention under drought than PI 398881 and PI 561370 (Supplementary Fig. S4B) and had a better canopy wilting visual score (Supplementary Fig. S2). The shoot physiological parameters such as stomatal conductance, internal CO_2_ concentration, and canopy temperature showed significant variation at both genotype and treatment interaction levels (Supplementary Table S5, available at *JXB* online). In general, the observed leaf area was reduced under water-limited conditions; the greatest reduction (36.6%) occurred in the exotic PI 427136 and the lowest reduction (0.3%) occurred in LD00-3309. PI 427136 also showed a reduction in stomatal conductance (Table 1) with an increase in canopy temperature under water-limited conditions. Unexpectedly, an increase in leaf area under water limitation was observed in three genotypes (LG04-4717, LD02-4485, and Maverick). Prohio, S06-13640, and 4J105-3–4 exhibited a 20.0%, 6.0%, and 0.3% increase in their photosynthetic rates under water-limited conditions, respectively, accompanied by reductions in other traits associated with stomatal regulation such as conductance (Fig. 3) and leaf canopy temperature. These genotypes showed higher internal CO_2_ concentrations available for carbon fixation under water stress. In this study, the genotypes varied widely for pod retention under drought stress, from 80.5 pods/plant (*n* = 6) in TN05-3027 to 24 pods/plant in LG05-4832 (Fig. 3).

**Table 1.** Summary of ANOVA for pod retention and number of nodes bearing pods in FT1 under water limitation

Trait	Mean	Maximum	Minimum	Genotype	Replication	Genotype × environment
Pod number	41.6	80.5	24	3.37***	0.01	101.28***
Nodes with pods	11.7	26.5	7.7	1.75**	2.68*	40.76***

* *P*-value of 0.1–0.05; ** *P*-value of 0.05–0.001; *** *P*-value of 0.001–0.0001

### Relationships among root anatomy and plant performance in water limitation

**Fig. 4.**
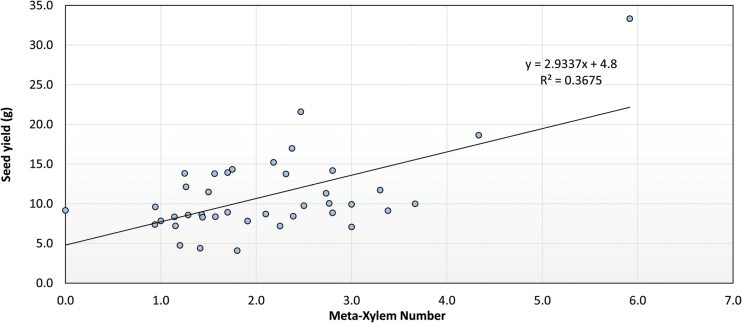
Regression analysis to show association between seedling metaxylem number and seed yield in NAM soybean lines screened in FT1.

A positive relationship existed between the seedling MX and seed yield in FT1 (R^2^ = 0.37, *P* < 0.05) (Fig. 4). MX also showed a positive association with the internal CO_2_ concentration (R^2^ = 0.35, *P* < 0.05) and stomatal conductance (R^2^ value = 0.38, *P* < 0.05) in drought conditions. The increase in MX was negatively correlated with canopy temperature (R^2^ = −0.34, *P* < 0.05) with an increase in water uptake. These shoot and root phenes enable the genotypes to maintain water uptake and water-use efficiency under water-limited conditions. In addition, the seedling root cortex cell area (CCA) (R^2^ = 0.82, *P* < 0.05) and percentage of cross section that is cortical cells (R^2^ = 0.60, *P* < 0.05) were positively correlated with seed yield on a single plant basis in FT1.

### Translation of resource-use efficiency into seed yield in rain-fed conditions

In FT2, genotypic variation existed for the percentage of total nitrogen (N) and carbon (C), and for their assimilation into the plant system as isotopes, N^15^ and C^13^, under water-deficit conditions that impacted the grain yield (Table 2). However, leaf total nitrogen and C^13^ showed significant differences according to treatment (irrigated versus rain-fed) Supplementary Fig. S5, available at *JXB* online). Under rain-fed conditions, the elite line LG05-4464 showed a 5.7% reduction in total biomass, which resulted in the highest recorded yield reduction of 24.2% and the largest recorded decrease in the percentage pod harvest index (40.7%). A few genotypes showed an increase in the pod harvest index under water-stress conditions, with the genotype HS6-3976 showing an increase of ~20% (Supplementary Fig. S6, available at *JXB* online). Interestingly, the high-yielding elite soybean genotype Skylla was found to have high water-use efficiency with lower stable isotope ratio (d13C) values, followed by those of LG04-6000, NE3001, and LG94-1906, than PIs screened in this study. However, PIs 398881 and 561370 were found to use water more efficiently in well-watered conditions than the elite lines. The d13C values that represents the water-use efficiency of all other lines used in this study are shown in Supplementary Fig. S5.

**Table 2.** Summary of ANOVA for yield (FT1, 2, and 3), carbon isotope discrimination value (Δ15 C), and nitrogen isotope discrimination value (Δ15 N) (FT2 and 3) for stress treatment

Trait	Mean	Maximum	Minimum	Genotype	Replication	Genotype × environment
Single plant yield (g)	11.2	33.3	4.1	1.32*	4.34**	122.75***
FT2 yield (g)	470.6	664.3	253.4	3.40***	10.63**	9.46**
FT3 yield (g)	530.7	816.5	276.7	1.10	3.62**	3.07**
Δ15 C Leaf	−28.5	−26.9	−29.8	1.39*	0.84	93.44***
Δ15 N Leaf	1.38	5.1	0.1	1.14	15.39***	84.57***

* *P*-value of 0.1–0.05; ** *P*-value of 0.05–0.001; *** *P*-value of 0.001–0.0001

### Detailed physiological characterization of genotypes with contrasting yields under drought stress

We examined genotypes that differed in constitutive root traits (in GHT1), yield performance under water-limited environments (FT1 and 2), and yield responses to reproductive phase stress. We selected three lines from each of the contrasting yield groups; the HYS group was represented by 4J105-3–4 (HYS-1), CL0J173-6–8 (HYS-2), and PI427136 (HYS-3), and the LYS group was represented by PI398881 (LYS-1), PI507681B (LYS-2), and PI561370 (LYS-3).

To gain further insight into the role of root anatomical traits in water uptake and transport towards the maintenance of normal physiological processes in the shoot and yield protection under water limitation, GHT3 was conducted. Twenty-eight days of drought stress was imposed on the selected lines in a mesocosm, permitting greater environmental control and detailed measurements of shoot and root trait plasticity. The HYS lines showed a higher percentage reduction in their photosynthetic rates in both field and rain-out shelter water-stress conditions with the exception of HYS-1, which showed an increase in its photosynthetic rate of approximately 0.3% and 2% with no significant variation in canopy temperature in FT1 and GHT3, respectively. This line was found to maintain a balance between transpiration loss and water uptake to meet the plant’s demands under water-limited conditions. The reduction in photosynthetic rate and internal CO_2_ was highly pronounced in HYS-2 and HYS-3, with a significant reduction in stomatal conductance (Fig. 3). The LYS lines showed less of a reduction in photosynthesis in both GHT3 and FT1 under water-limited conditions with the exception of LYS-3, which showed the largest reduction in photosynthesis in GHT3 and the largest reduction in conductance and internal CO_2_ capture in both GHT3 and FT1 under water-limited conditions. Although these experimental locations differed in environmental factors, such as the intensity of drought stress, the LYS had poor water-use efficiency values (lowest ^13^C values from FT2), poor maintenance of their photosynthetic rates, and poor carbon fixation efficiency, resulting in poor grain yield in stressful environments (FT1, 2, and 3).

### Modulation of xylem number and diameter enhances water uptake during drought stress

**Fig. 5.**
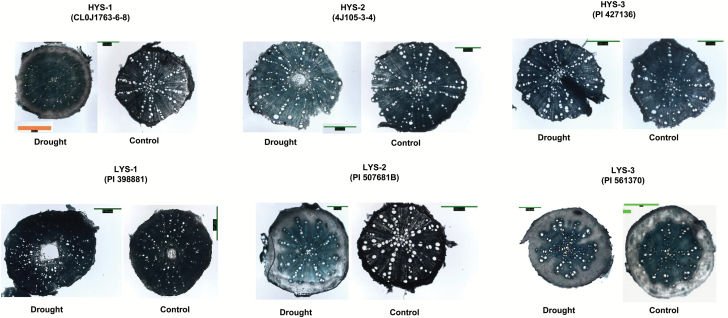
Variation in root anatomical response observed in metaxylem and cortex cell area among HYS and LYS lines under irrigated and drought conditions. Scale barS: HYS-1 Drought (1564 μm) and Control (715 μm); HYS-2 Drought (1001 μm) and Control (715 μm); HYS-3 Drought (1001 μm) and Control (715 μm); LYS-1 to 3 Drought (715 μm) and Control (1001 μm).

The lines selected for the mesocosm experiment in GHT3 demonstrated drought adaptation by altering the root anatomical traits RXSA, TCA, and MX. The three HYS lines showed a reduction in RXSA with an increase in MX, which is considered a common response to drought conditions (Fig. 5). All LYS lines showed the opposite response with respect to these anatomical traits except for the MX of LYS-1 (Fig. 5), which resulting in LYS-1 having the highest root hydraulic conductivity (RHC) under drought conditions of the six lines (Supplementary Fig. S7, available at *JXB* online). The LYS-3 RHC and shoot hydraulic conductivity followed similar trends to those of HYS lines under drought (Supplementary Fig. S7). The relationship between average RHC and MX showed a regression (R^2^) value of 0.4. The alterations to the root anatomical structures (RXSA and MX) in LYS could have affected water transport and imposed limitations on leaf conductance, which in turn could have affected the carbon fixation efficiency under water-limited conditions. The xylem elements in HYS lines had smaller diameters (Supplementary Fig. S8, available at *JXB* online) than those in the LYS lines, except LYS2. The changes in RHC were highly associated with xylem number and diameter. In contrast, the reduction in RXSA (positively correlated with lateral root length in GHT 1) and increase in MX in the HYS lines facilitated the metabolic cost required to explore deeper soil strata and to enhance water transport, allowing the maintenance of normal shoot physiological processes, which led to yield protection in water-limited environments.

## Discussion

Several studies (Meister *et al.*, 2014; Rogers and Benfey, 2015) emphasize the significance of root modifications for higher grain yields in nutrient- and water-limited soils and for increased stress tolerance in crop plants. The importance of root traits has been known for decades, but their genetic nature and interactions with other external factors affected their suitability for selection in field conditions (Prince *et al.*, 2013). Most root studies in soybean have been focused on the seedling stage to identify genomic regions associated with different root architectural traits (Liang *et al.*, 2010; Zhou *et al.*, 2011; Brensha *et al.*, 2012; Liang *et al.*, 2014; Manavalan *et al.*, 2015; Prince *et al.*, 2015), and a few studies have also been carried out in field conditions at the reproductive stages (Abdel-Haleem *et al.*, 2011; Prince *et al.*, 2016). The present study with the SoyNAM panel of high-yielding lines (32 elite lines, 8 PIs, and a hub parent, IA3203) revealed that high phenotypic variation in seedling root length and the anatomical trait MX was associated with yield under drought. Interestingly, the high-yielding exotic PI 427136 has a reduced leaf area that helps to conserve water and yield better during drought-stress conditions. A similar reduction in leaf area has been documented in different crop species (Nautiyal *et al.*, 2002; Kadam *et al.*, 2015) as a mechanism of water conservation. The combined results from our mesocosm and field trials provide evidence to support our conclusions that root architecture and anatomical traits contribute to enhanced above-ground performance in the HYS lines by maintaining photosynthesis and stomatal regulation, resulting in better yield under water-stress conditions.

At the leaf level, the genotype-specific efficiency of carbon and nitrogen assimilation play a key role in determining the final seed yield in water-limited environments. The C^13^ and total leaf nitrogen content could potentially be used as selection indices to identify genotypes with enhanced resource-use efficiency. To maintain a higher seed yield in both optimal and suboptimal environments, nitrogen accumulation must be maintained in leaves to achieve adequate biomass for the balance in the source–sink relationship (Nobuyasu *et al.*, 2003; Van Roekel and Purcell, 2014). In many crop species, carbon isotope discrimination values have been demonstrated as simple and reliable measurements of water-use efficiency (Condon *et al.*, 1993; Xu *et al.*, 2009; This *et al.*, 2010), and they are utilized as key selection indices in breeding programmes to select for increased water-use efficiency and yield under rain-fed conditions (Blum 1996; Richards 1996; Richards *et al.*, 2002).

Further identification and utilization of novel alleles from the high-yielding exotic line PI 427136 could improve root architectural traits, such as root length, projection, and surface area. The low prevalence of these traits in the high-yielding germplasm could be due to the selective pressures of increased planting densities. Similar narrowing in the phenotypic variability of shoot and root architectures has been observed in wheat cultivars because of higher planting densities (Hammer *et al.*, 2009). The PI 427136 was also reported to harbour quantitative trait loci for yield under water-limited conditions, and had improved yielded in both well-watered and water-limited conditions compared with other two NAM population members tested in 12 US locations (Gibson, 2015). Thus, alleles introgressed from the high-yielding exotic PI 427136 into other elite lines have the potential to improve nutrient and water conservation, resulting in lower yield penalties under water limitation.

Various root anatomical phenes have been reported to be associated with plant performance and productivity under water-limited conditions in several cereal crops (Chimungu *et al.*, 2014*a*, *b*; Chimungu *et al.*, 2015*b*; Kadam *et al.*, 2015) and a few in legume crops (Peña-Valdivia *et al.*, 2010; Purushothaman *et al.*, 2013). To the best of our knowledge, none of the previous studies correlated reduced MX with enhanced yield performance under drought stress. These water-conducting xylem elements play a vital role not only in the uptake but also in the transport of nutrients and water within the plant system (Steudle and Peterson, 1998). In a recent study, Kadam *et al.* (2015) demonstrated the plasticity of root elements in wheat: stele and xylem properties are key traits that improve water-use efficiency in wheat over that in rice. This study has emphasized the need to establish the relationships between root morphological and anatomical trait plasticity and yield under stress in soybean. The present study has established the relationship between the plasticity of MX and xylem diameter with improved yield under water-deficit stress. Other root anatomical trait such as CCS may also play a beneficial role in enhancing yield under drought stress. A study by Chimungu *et al.* (2014*a*) showed that maize lines with increased CCS had improved soil exploration, water acquisition, growth, and development under drought. Therefore, more study is required to provide further validation with soybean lines with contrasting CCA and to clarify the relationship of CCA with enhanced yield.

We conclude that the MX is an acceptable proxy for stomatal regulation and capture of CO_2_ in leaves, allowing for easier selection of genotypes with enhanced water/nutrient uptake and use efficiency, resulting in enhanced RHC and higher seed yield in HYS lines under water-limited conditions. MX and xylem diameter are easily observable traits in mature soybean plants under water-limited conditions, even with the advancement of secondary thickening (Purushothaman *et al.*, 2013). Legume crop genotypes with adaptation mechanisms such as the alteration of xylem vessels (number and size) and cortex cells can enhance productivity under drought stress without incurring a yield penalty under irrigated conditions. Selecting high-yielding lines based on xylem diameter has previously proven successful in a drought breeding programme for wheat (Richards and Passioura, 1981*a*, 1981*b*, 1989).

The three HYS lines identified in this study within the soybean NAM panel could facilitate the development of soybean lines with different drought-adaptive mechanisms to stabilize the seed yield under water-limited conditions. PI 427136 among the HYS group exhibits superior constitutive seedling root architectural traits and a reduction of leaf area, allowing for efficient water uptake and use during periods of water limitation. Similar modulation of shoot and root traits has been reported previously for other exotic soybean PIs (Prince *et al.*, 2016). Interestingly, the mapping population generated by all of the HYS individuals and the hub parent IA3203 had higher yielded than other crosses did across several locations in the US differing in soil and climatic factors (Gibson, 2015). Thus, these three HYS genotypes should be used as the breeding material to narrow the yield gap in soybean (Grassini *et al.*, 2014*a*; Grassini *et al.*, 2015) by improving water uptake and use efficiency in both rain-fed and irrigated crops. The NAM population developed with these lines would provide valuable genetic material to map yield and yield-related components in water-limited environments. In addition to the elite lines (HYS1 and 2), the exotic PI 427136 (HYS3) line will bring novel alleles to the elite germplasm to enhance productivity in rain-fed soybean-growing environments.

## Conclusions

Modulating leaf and root adaptive mechanisms is essential to protect yield during drought stress. We propose that the selection of the key seedling root anatomical trait, MX, would facilitate the selection of genotypes suited for both optimum and water-deficit environments. Plasticity in MX number and xylem diameter facilitate improved water-use efficiency and yield protection under water stress. The high-yielding genotypes we have identified to be suited to both terminal drought stress and receding soil moisture conditions will help the research community to narrow the yield gap in soybean in water-limited conditions.

## Supplementary data

Supplementary data are available at *JXB* online.

Table S1. Global root architectural traits of 41 NAM panel lines used in this study.

Table S2. Total monthly precipitation, irrigation, and average maximum daily temperature for the field studies conducted (FT 1, 2 and 3).

Table S3. Summary of ANOVA for constitutive seedling (V1 stage) root architectural traits.

Table S4. Phenotypic variation observed for seedling root anatomical traits in NAM panel.

Table S5. Summary of ANOVA for the effects of water limitation on shoot physiological traits in FT1 under drought-stress treatment.

Fig. S1. Depletion of soil moisture in the FT1 experiment measured using a Delta-T probe soil moisture sensor with a PR2 probe at four different depths varying from 300 to 1000 mm.

Fig. S2. Canopy wilting scores of HYS and LYS under drought in FT1.

Fig. S3. Variation in seedling root system architecture in (A) PI427136 and (B) LG00-3372.

Fig. S4. (A) Rainout shelter located at the Bradford Research and Extension Center used for FT1. (B) Variations in pod retention/load observed under drought conditions during FT1 between HYS-3 and LYS-1 and -3.

Fig. S5. Variation observed for water-use efficiency (based on C^13^ values) and total percentage nitrogen content in soybean leaf tissues (L) under irrigated (IR) and rain-fed (RF) conditions in FT2.

Fig. S6. Significant variation for percentage pod harvest index in the late reproductive stage of NAM soybean genotypes under water-limited conditions in FT2.

Fig. S7. Hydraulic conductivity (kg/s) for water-stressed roots (WSR), well-watered roots (WWR), water-stressed shoots (WSS), and well-watered shoots (WWS) from GHT3 for HYS and LYS.

Fig. S8. Variation in plasticity of xylem diameter (mm) among HYS and LYS lines under drought stress.

## Supplementary Material

Supplementary_Figures_S1_S8

Supplementary_Table_S1

Supplementary_Table_S2

Supplementary_Table_S3_S5
