# Supplementary material for: Root xylem plasticity to improve water use and yield in water-stressed soybean
Source: J Exp Bot. 2017 Jan 7;68(8):2027–36. doi: 10.1093/jxb/erw472 (PMC5428998; doi:10.1093/jxb/erw472)
Supplement: Supplementary_Figures_S1_S8 [file erw472_suppl_Supplementary_Figures_S1_S8.pdf]

Root xylem plasticity to improve water use and yield in water-stressed soybean

Silvas J. Prince, Mackensie Murphy, Raymond N. Mutava, Lorellin A. Durnell, Babu Valliyodan, J. Grover Shannon, Henry T Nguyen

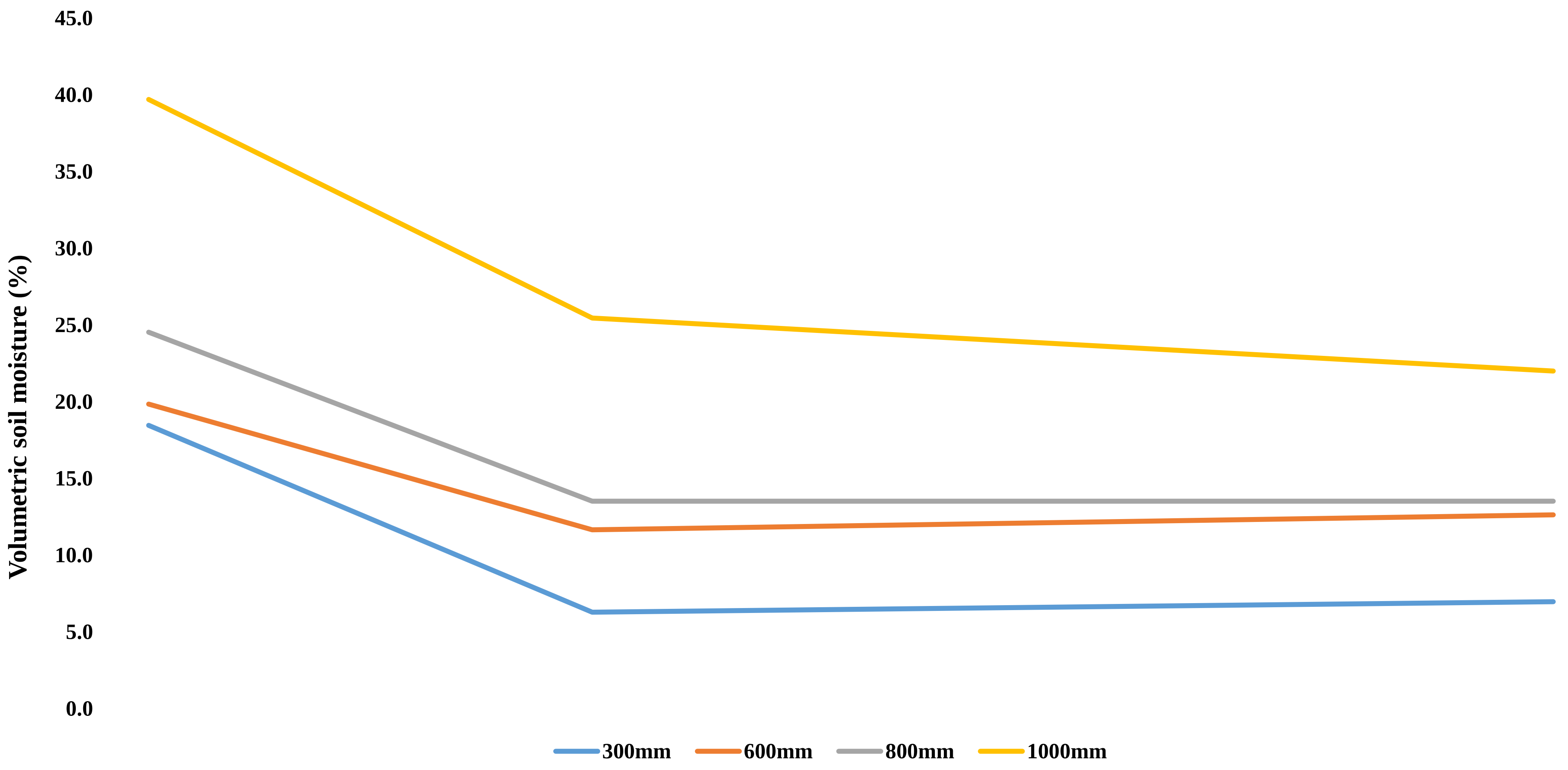

Figure S1. Depletion of soil moisture in FT1 experiment measured using Delta T probe soil moisture sensor with PR2 probe at four different depths varying from 300-1000mm

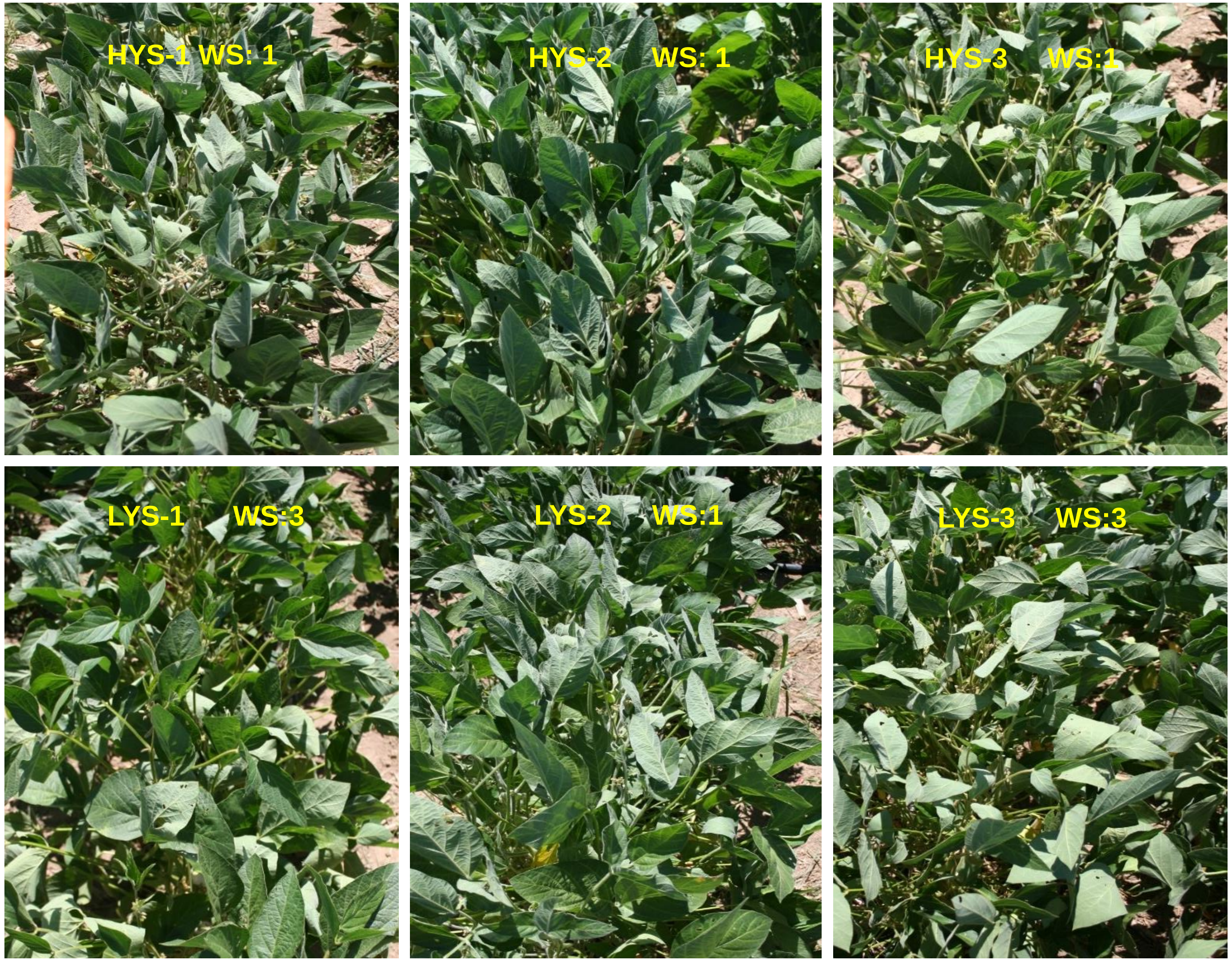

Figure S2. Canopy wilting scores (WS) of HYS and LYS under drought in FT1.

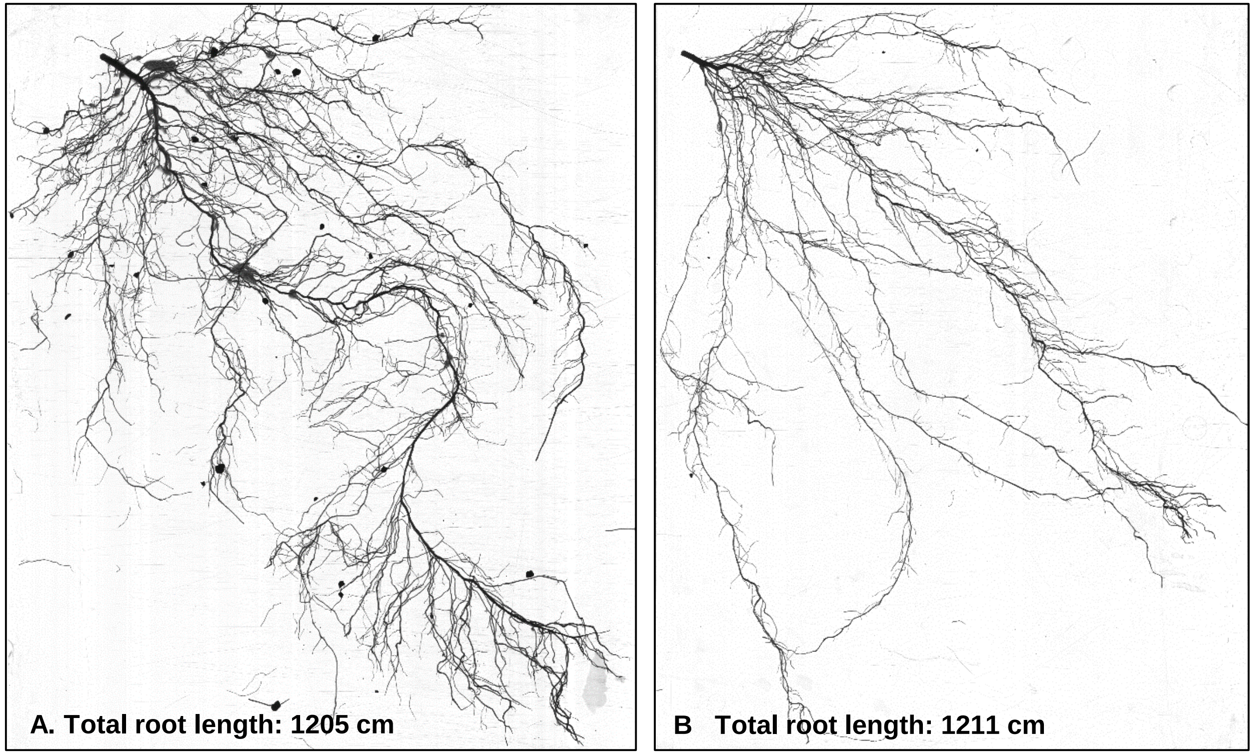

Figure S3. Variation in seedling root system architecture in (A) PI427136 and (B) LG00-3372

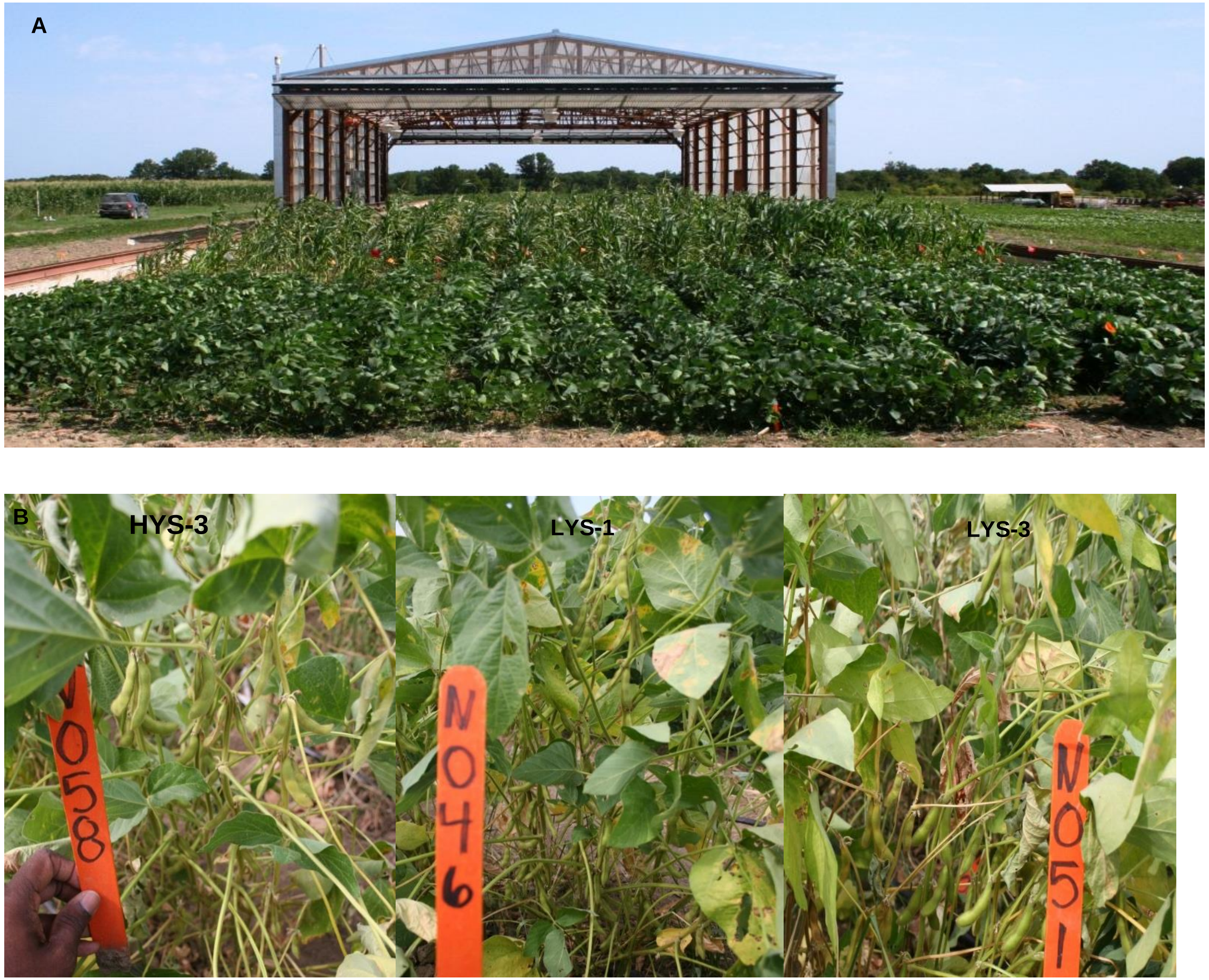

Figure S4. (A) Rain out shelter located at Bradford Research and Extension Center (BREC) Columbia, Missouri used for FT1. (B) Variations in pod retention/load observed under drought conditions during FT1 between HYS-3 and LYS-1 and -3.

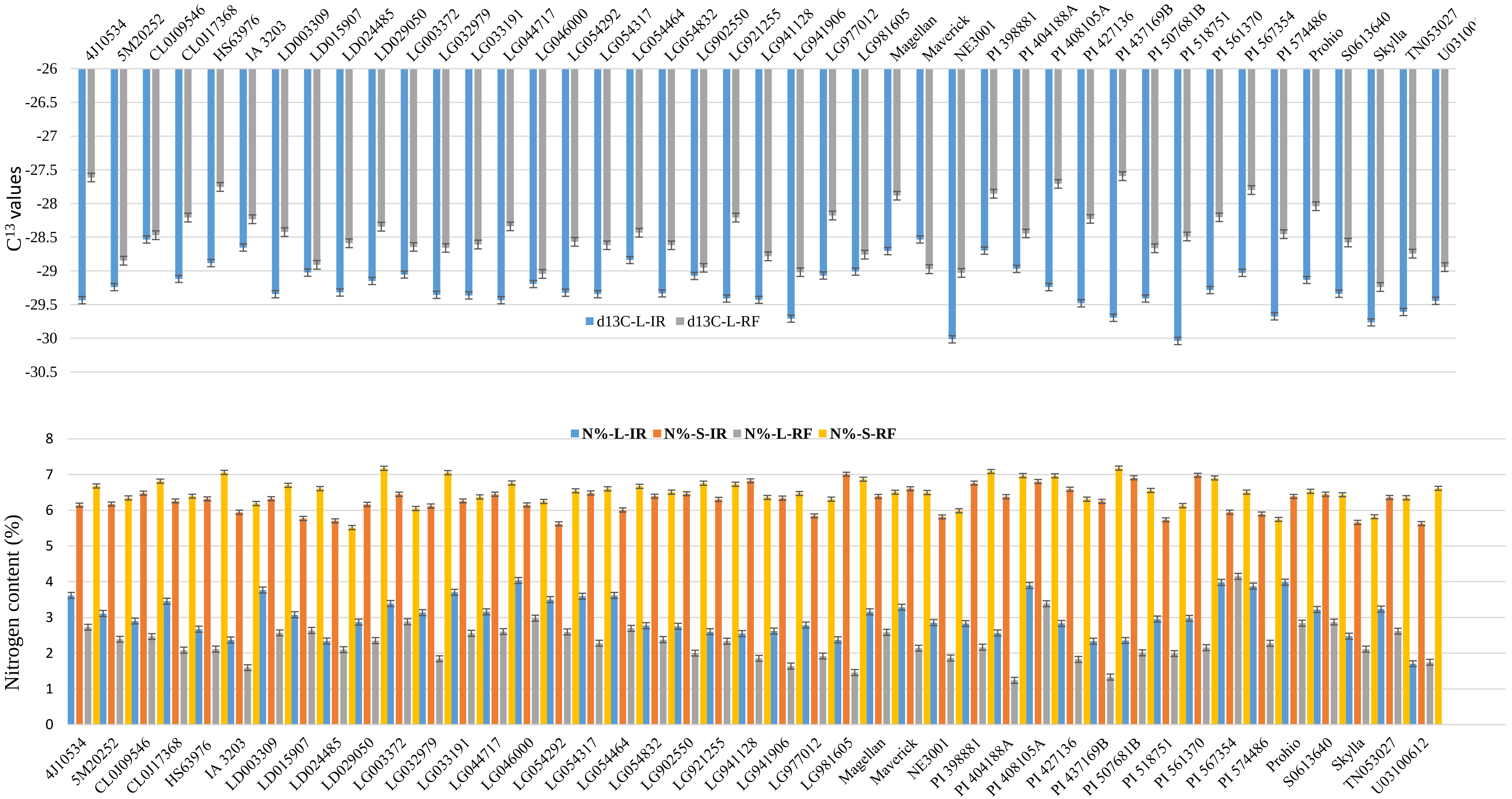

Figure S5. Variation observed for water-use efficiency (based on C^{13}values) and total percent nitrogen content in soybean leaf tissues (L) under irrigated (IR) and rainfed (RF) conditions in FT2

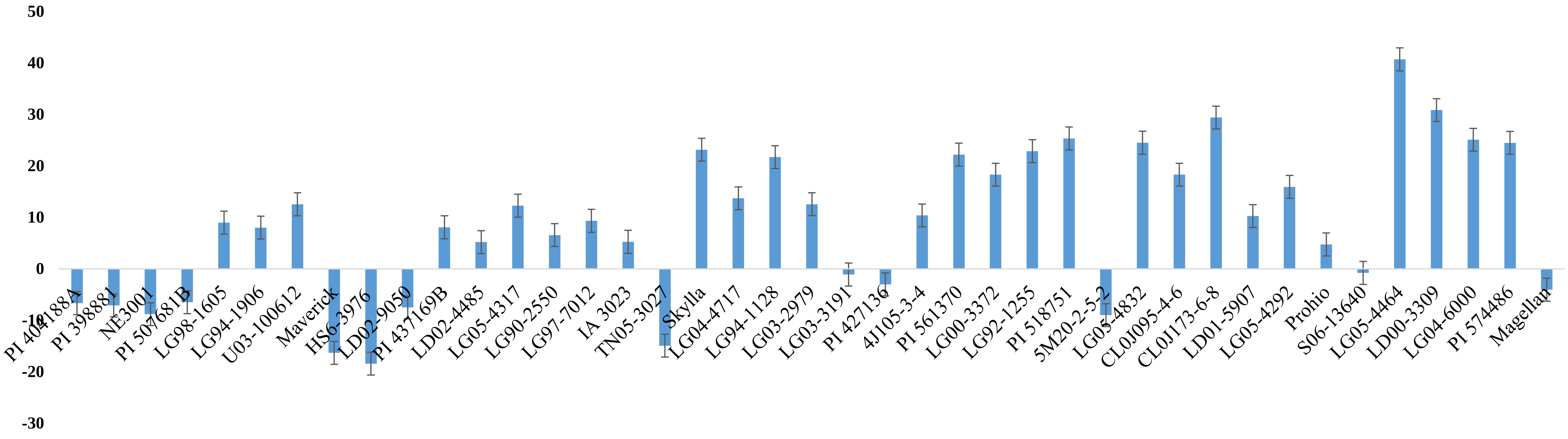

Figure S6. Significant variation for percentage pod harvest index in the late reproductive stage of NAM soybean genotypes in water-limited condition of FT2. Positive and negative values of Y-axis represents decrease and increase in pod harvest index, respectively.

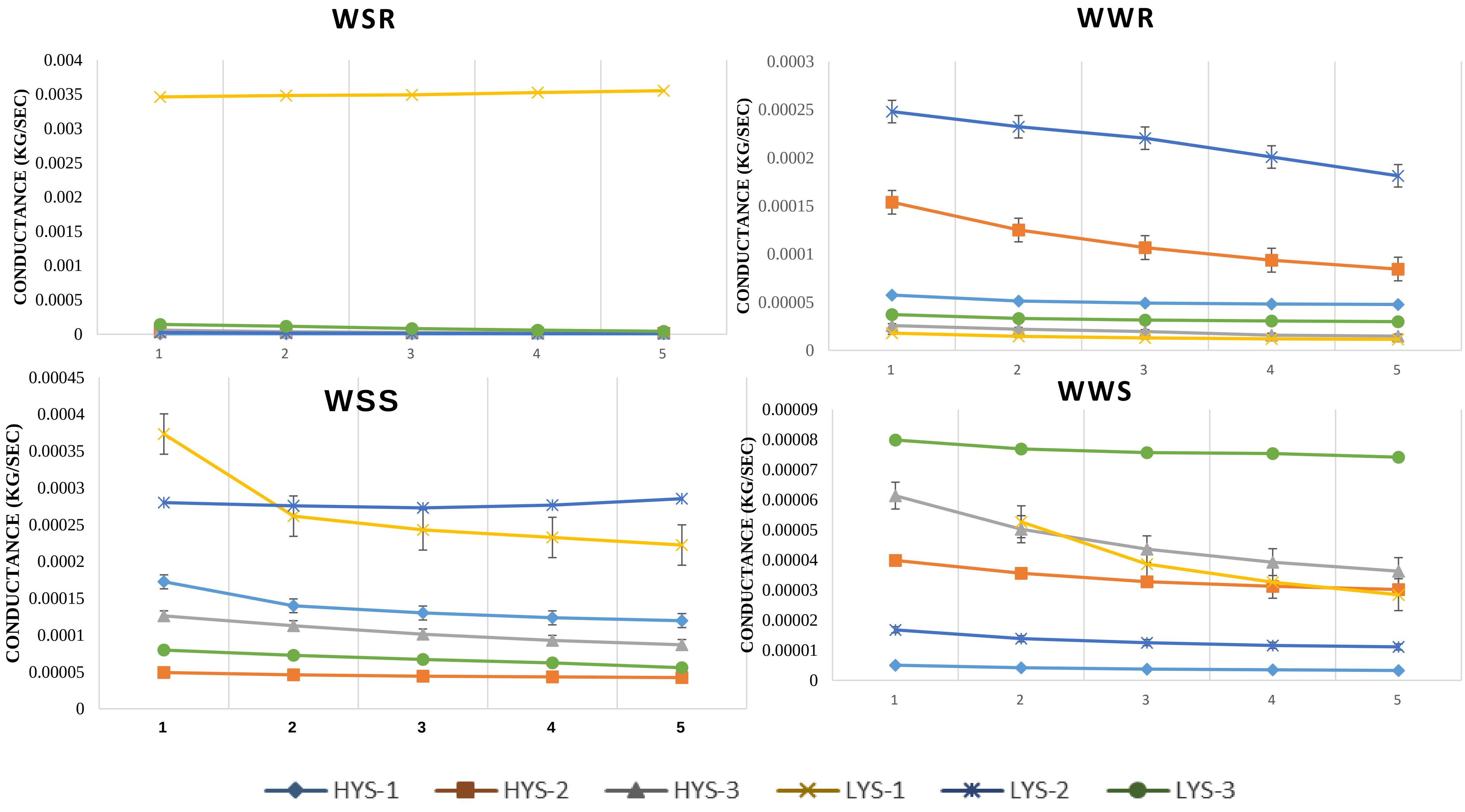

Figure S7. Hydraulic conductivity (Kg/s) for water-stressed roots (WSR), well-watered roots (WWR), water-stressed shoots (WSS), and well-watered shoots (WWS) from GHT3 for HYS and LYS

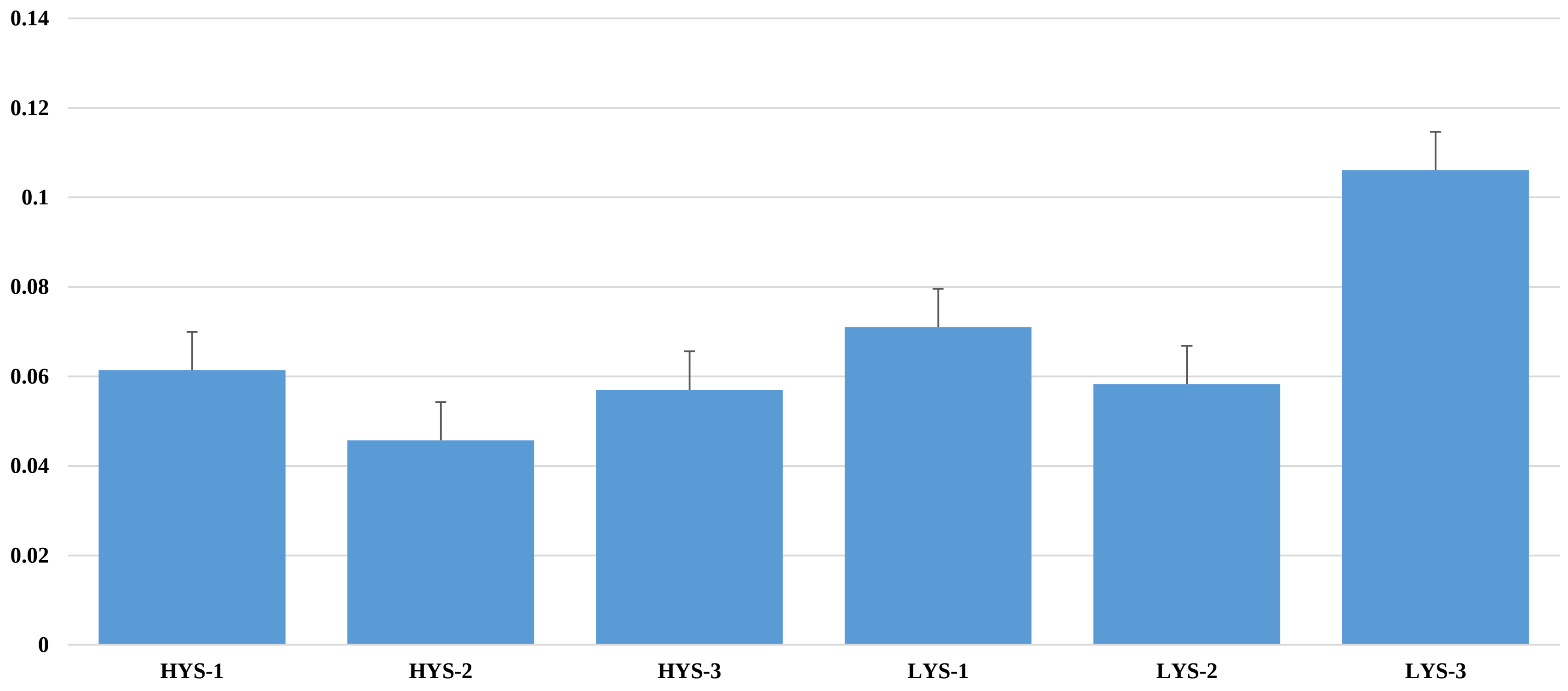

Figure S8. Variation in plasticity of xylem diameter (mm) among HYS and LYS lines under drought stress
