# Supplementary material for: Root xylem plasticity to improve water use and yield in water-stressed soybean
Source: J Exp Bot. 2017 Jan 7;68(8):2027–36. doi: 10.1093/jxb/erw472 (PMC5428998; doi:10.1093/jxb/erw472)
Supplement: Supplementary_Table_S3_S5 [file erw472_suppl_Supplementary_Table_S3_S5.pdf]

Table S3. Summary of ANOVA for constitutive seedling (V1 stage) root architectural traits

Trait	Mean	Maximum	Minimum	Genotype	Replication
Total root length (L)	946.9	1210.9	536.4	4.73***	4.45**
Number of Crossings (C)	540.1	760.6	241.0	2.86***	2.37*
Root Volume (RV)	1.3	1.6	0.7	3.82***	1.18
Number of Forks (F)	2632.4	3703.8	1142.3	2.99**	2.01
Number of Tips (T)	1478.8	2985.0	812.0	2.82***	2.01
Taproot Length (TL)	35.4	39.1	28.4	0.98	1.48
Projected Area (PA)	39.2	49.3	22.0	4.96***	2.94**
Surface Area (SA)	123.2	155.0	69.2	4.96***	2.94**

* *P*-value of 0.1 to 0.05, ** *P*-value of 0.05 to 0.001, *** *P*-value of 0.001 to 0.0001

Table S4. Phenotypic variation observed for seedling root anatomical traits in NAM panel

Trait	Mean	Maximum value	Minimum value	Genotype	Replication
Root cross-sectional area (RXSA)	0.437	0.821	0.131	1.31*	1.00
Total cortex area (TCA)	0.453	0.709	0.105	1.36*	0.95
Cortical cell number (CN)	371.562	674.800	96.00	1.27	0.75
Cortical cell file number (CCFN)	8.415	11.7	4	2.39***	0.99
Total stele area (TSA)	0.151	3.488	0.026	1.17	1.34
Percent of cross section is meta xylem (PXSMX)	2.332	8.182	0.00	4.63***	0.29
Number of metaxylem elements (MX)	2.105	5.917	0.000	3.91***	0.23
Cortex cell area (CCA)	0.485	13.126	0.019	2.22**	0.76
Meta xylem vessel area (MXVA)	0.053	2.063	0.00	2.02**	0.51
Percent of cross section is cortical cells (PXSCC)	32.829	59.494	14.586	14.59***	0.40
Percent of cortex that is cortical cells (PCCC)	33.774	45.2	18.213	11.23***	0.33

* *P*-value of 0.15 to 0.05, ** *P*-value of 0.05 to 0.001, *** *P*-value of 0.001 to 0.0001

Table S5. Summary of ANOVA for the effects of water limitation on shoot physiological traits in FT1 under drought stress treatment

Trait	Units	Mean	Max	Min	G	Rep	G x E
Stomatal conductance (SC)	mmol m^{-2} s^{-1}	0.18	0.39	0.05	0.91	1.61	419.00***
Leaf area (LA)	cm^2	33.10	45.00	15.90	5.02***	12.93***	99.58***
Internal CO_2 (Ci)	µmol $mol^{-)}$	151.85	192.30	110.54	0.72	1.38	394.486***
Canopy Temperature (CT)	Degrees C	35.78	38.41	32.27	0.85	2.17	116.49***
Photosynthetic Rate (Photo)	-	11.28	18.48	4.34	1.21	3.06*	148.10***
Plant height (Ph)	cm	70.00	103.20	46.70	8.74***	0.06	347.29***

* *P*-value of 0.1 to 0.05, ** *P*-value 0.05 to 0.001, and *** *P*-value of 0.001 to 0.0001
